# Supplementary material for: A fault-intrusion-tolerant system and deadline-aware algorithm for scheduling scientific workflow in the cloud
Source: PeerJ Comput Sci. 2021 Nov 2;7:e747. doi: 10.7717/peerj-cs.747 (PMC8576554; doi:10.7717/peerj-cs.747)
Supplement: Supplemental Information 6 [file peerj-cs-07-747-s006.zip › Myproject1.1/docs/org/cloudbus/cloudsim/network/FloydWarshall_Float.html]

FloydWarshall\_Float


JavaScript is disabled on your browser.


- Overview
- Package
- Class
- Use
- Tree
- Deprecated
- Index
- Help

- Prev Class
- Next Class

- Frames
- No Frames

- All Classes

- Summary:
- Nested |
- Field |
- Constr |
- Method

- Detail:
- Field |
- Constr |
- Method


org.cloudbus.cloudsim.network

## Class FloydWarshall\_Float

- java.lang.Object
- - org.cloudbus.cloudsim.network.FloydWarshall\_Float

- ---

    

  ```
  public class FloydWarshall_Float
  extends java.lang.Object
  ```

  FloydWarshall algorithm to calculate all pairs delay and predecessor matrix.

  Since:
  :   CloudSim Toolkit 1.0

- - ### Constructor Summary

    Constructors

    Constructor and Description

    `FloydWarshall_Float()`
  - ### Method Summary

    Methods

    | Modifier and Type | Method and Description |
    | --- | --- |
    | `float[][]` | `allPairsShortestPaths(float[][] adjMatrix)` calculates all pairs delay |
    | `int[][]` | `getPK()` gets predecessor matrix |
    | `void` | `initialize(int numVertices)` initialization matrix |

    - ### Methods inherited from class java.lang.Object

      `clone, equals, finalize, getClass, hashCode, notify, notifyAll, toString, wait, wait, wait`

- - ### Constructor Detail


    - #### FloydWarshall\_Float

      ```
      public FloydWarshall_Float()
      ```
  - ### Method Detail


    - #### initialize

      ```
      public void initialize(int numVertices)
      ```

      initialization matrix

      Parameters:
      :   `numVertices` - number of nodes


    - #### allPairsShortestPaths

      ```
      public float[][] allPairsShortestPaths(float[][] adjMatrix)
      ```

      calculates all pairs delay

      Parameters:
      :   `adjMatrix` - original delay matrix

      Returns:
      :   all pairs delay matrix


    - #### getPK

      ```
      public int[][] getPK()
      ```

      gets predecessor matrix

      Returns:
      :   predecessor matrix


- Overview
- Package
- Class
- Use
- Tree
- Deprecated
- Index
- Help

- Prev Class
- Next Class

- Frames
- No Frames

- All Classes

- Summary:
- Nested |
- Field |
- Constr |
- Method

- Detail:
- Field |
- Constr |
- Method
